# Meiotic arrest with roscovitine and follicular fluid improves cytoplasmic maturation of porcine oocytes by promoting chromatin de-condensation and gene transcription

**DOI:** 10.1038/s41598-017-11970-y

**Published:** 2017-09-14

**Authors:** Min Zhang, Chuan-Xin Zhang, Liu-Zhu Pan, Shuai Gong, Wei Cui, Hong-Jie Yuan, Wei-Ling Zhang, Jing-He Tan

**Affiliations:** College of Animal Science and Veterinary Medicine, Shandong Agricultural University, Tai-an City, 271018 P.R. China

## Abstract

The developmental capacity of *in vitro* matured oocytes is inferior to that of the *in vivo* matured ones due to insufficient cytoplasmic maturation. Although great efforts were made to accomplish better cytoplasmic maturation by meiotic arrest maintenance (MAM) before *in vitro* maturation (IVM), limited progress has been achieved in various species. This study showed that MAM of porcine oocytes was better achieved with roscovitine than with dibutyryl cyclic adenosine monophosphate (db-cAMP) or 3-isobutyl-1-methylxanthine. Oocyte developmental competence after IVM was significantly improved following MAM in 199 + FF medium (TCM-199 containing 10% porcine follicular fluid and 25 µM roscovitine) to a level even higher than that in control oocytes matured without pre-MAM. Observations on other markers further confirmed the positive effects of MAM in 199 + FF on oocyte cytoplasmic maturation. During MAM culture in 199 + FF, re-decondensation (RDC) of condensed chromatin occurred, and transcription of genes beneficial to cytoplasmic maturation was evident in some of the oocytes with surrounded nucleoli (SN). However, MAM with db-cAMP neither induced RDC nor improved oocyte developmental potential. Together, the results suggest that MAM in the presence of FF and roscovitine improved the developmental competence of porcine oocytes by promoting a pre-GVBD chromatin de-condensation and expression of beneficial genes.

## Introduction

It has been demonstrated in various species that the developmental capacity of *in vitro* matured (IVM) oocytes is inferior to that of the *in vivo* matured oocytes. It is known that oocytes must undergo both nuclear maturation and cytoplasmic maturation to gain the capability to support successful fertilization and embryo development^1^. *In vivo*, oocytes acquire cytoplasmic maturity after a long series of strictly-controlled preparatory processes involving transcription and subsequent translation of transcripts during the meiotic prophase^1^. *In vitro*, however, a sudden premature meiotic resumption without adequate cytoplasmic maturation is induced by transfer of oocytes from follicles into the culture medium. Thus, it was hypothesized that meiotic arrest maintenance (MAM) *in vitro* before IVM would allow oocytes more time to accomplish a better cytoplasmic maturation.

Although many studies were conducted to improve oocyte developmental competence through a pre-IVM MAM, limited progress has been achieved in various species. For example, the most commonly reported results following *in vitro* MAM of porcine^2^ and bovine^3^ oocytes were the obtainment of blastocyst rates similar to those obtained with control, non-MAM oocytes after IVM. However, there are indeed papers reporting promising results. For example, one paper reported that exposure of porcine oocytes to dibutyryl cyclic adenosine monophosphate (db-cAMP) for the first 20 h of IVM significantly improved the homogeneity of oocyte nuclear maturation and increased the blastocyst rates after *in vitro* fertilization^4^. Another paper reported higher blastocyst rates when bovine oocytes were initially arrested by inhibiting the maturation-promoting factor (MPF) with butyrolactone I in the presence of fetal bovine serum in 5% O_2_ compared to when oocytes were matured directly following follicle aspiration^5^. Recently, studies have achieved significant improvement of oocyte competence by elevating cAMP levels prior to IVM in mice^6, 7^, human^7^ and bovine^8^, and oocyte maturation and blastocyst development were significantly improved after MAM of immature oocytes from juvenile mice using C-type natriuretic peptide (CNP)- supplemented medium containing FSH and growth and differentiation factor-9^9^. However, the mechanisms by which the *in vitro* MAM improves oocyte competence are largely unknown.

Although cAMP modulation delayed meiotic progression of sheep IVM oocytes, it did not improve their developmental competence^10^. Treatment with cAMP could keep meiotic arrest for only a limited time in bovine^11^ and goat^12^ oocytes. Furthermore, several studies point to important differences between oocytes from rodent and livestock species in the mechanisms for oocyte meiotic arrest and resumption^13^. Thus, there might be species differences in oocyte response to different MAM protocols. In addition, while Funahashi *et al*.^4^ observed a significant increase in the blastocyst rates after exposing prepubertal porcine oocytes to db-cAMP for the first 20 h of IVM, Bagg *et al*.^14^ reported that db-cAMP treatment for the first 22 h of IVM had no effect on the subsequent blastocyst formation of adult pig oocytes. Together, the data suggest an urgent demand for more efficient MAM protocols for different species and for different type oocytes.

Furthermore, although the above reports suggest that oocytes show active metabolic activities during MAM *in vitro*, which can be used to improve their cytoplasmic maturation by manipulating the MAM nutrient conditions, studies on the ongoing cellular activities such as gene transcription and on nutrient requirements during *in vitro* MAM are very limited. In this study, we have explored the mechanisms by which MAM improves oocyte cytoplasmic maturation by examining the effects of follicular fluid (FF) and roscovitine/db-cAMP in the MAM media on chromatin configuration, gene transcription and cytoplasmic maturation of porcine oocytes. The results showed that a pre-IVM MAM in the presence of FF and roscovitine improved the developmental competence of adult porcine oocytes by promoting a pre-germinal vesicle (GV) breakdown (GVBD) chromatin de-condensation and transcription of beneficial genes.

## Results

### *In vitro* MAM of pig oocytes is better achieved with roscovitine than with db-cAMP or IBMX

To test the effects of the inhibitors often used for MAM, pig oocytes were incubated for 24 h in the α-MEM medium containing different concentrations of roscovitine, db-cAMP or IBMX before examination for GVBD. Whereas 96% of the oocytes were maintained at the GV stage following MAM culture with an optimal concentration (25 µM) of roscovitine, percentages of GV-intact oocytes (83–84%) were significantly lower after MAM with optimal concentrations (1 mM) of db-cAMP or IBMX (Supplementary Fig. S1). Furthermore, the MAM effect was less consistent using db-cAMP or IBMX than using roscovitine, as larger standard errors in the percentages of GV-intact oocytes were observed after MAM with db-cAMP or IBMX than with roscovitine. The results suggest that MAM of pig oocytes is better achieved by inhibiting MPF activation with roscovitine than by elevating cAMP levels with db-cAMP or IBMX. Thus, 25 µM roscovitine was used for MAM in most of the following experiments.

### Effects of MAM media on maturation and developmental potential of pig oocytes

**Figure 1:**
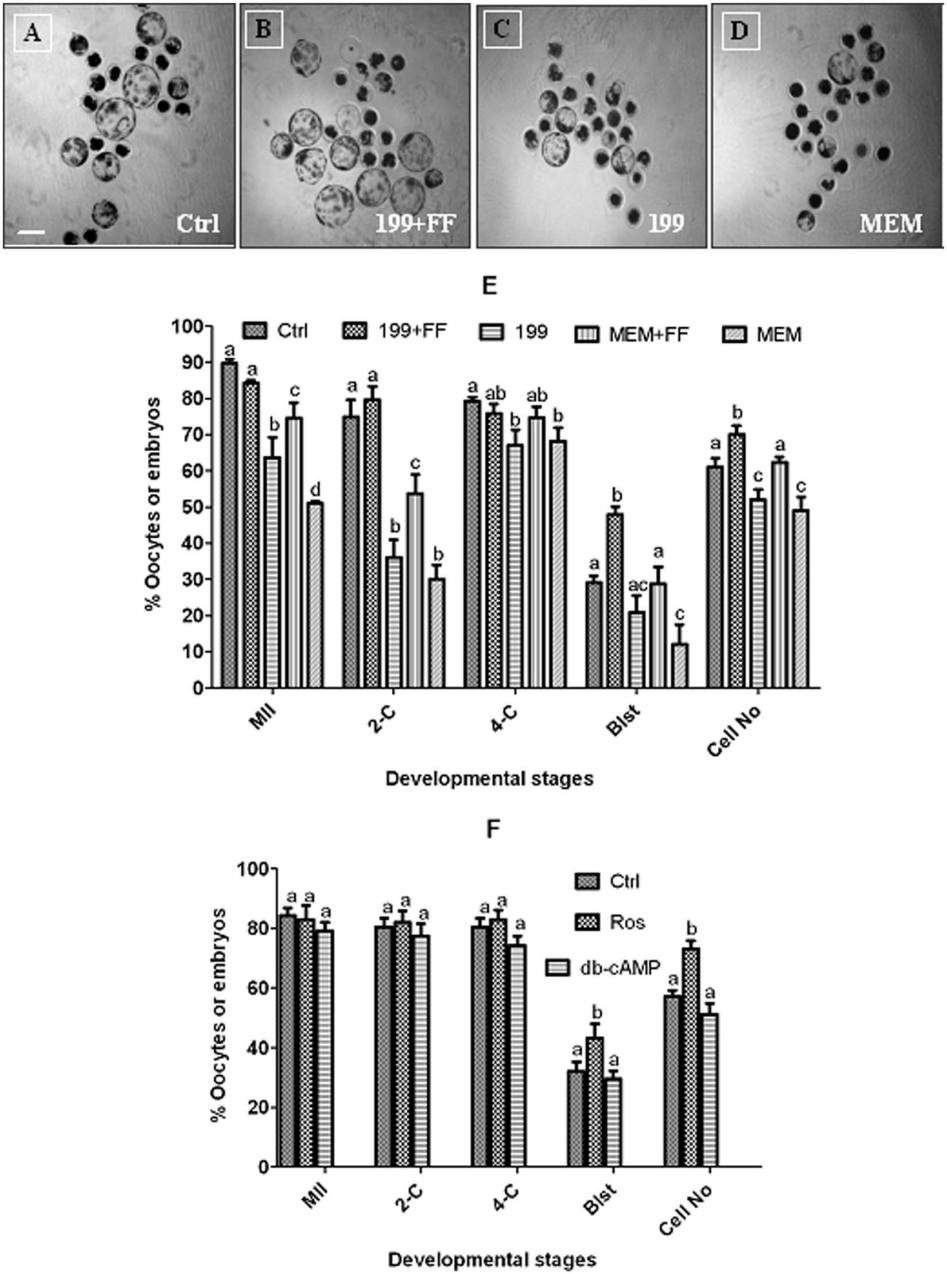
Parthenogenetic embryo development after pig oocytes were matured for 24 h following MAM for 24 h in different MAM media containing 25 µM roscovitine or 1 mM db-cAMP. Micrographs (**A**,**B**,**C** and **D**) show embryos recovered on day 7 of embryo culture in control oocytes matured for 48 h without MAM (Ctrl) and in oocytes matured for 24 h after MAM in 199 + FF, 199 or MEM, respectively. The bar is 200 µm. While graph E shows the effect of different MAM media containing 25 µM roscovitine, graph F shows the effects of roscovitine and 1 mM of db-cAMP contained in 199 + FF medium, on percentages of mature (MII)/cultured oocytes, 2-cell embryos/cultured oocytes, 4-cell embryos/2-cell embryos, blastocysts (Blst)/4-cell embryos, and cell number/blastocyst (Cell No), after parthenogenetic activation. Each treatment was repeated 4–5 times with each replicate including about 30–60 oocytes. a-c: Values without a common letter above bars differ significantly (P < 0.05) within developmental stages.

Oocytes were matured for 24 h following MAM for 24 h in different media containing 25 µM roscovitine. Control oocytes were matured for 48 h without MAM. At the end of maturation culture, some of the oocytes were freed of cumulus cells to observe first polar body extrusion, and others were activated with ionomycin and 6-DMAP for embryo development. Compared to those in control oocytes, percentages of MII oocytes, 2-cell and 4-cell embryos and blastocysts, and cell number per blastocyst, were significantly lower after MAM with TCM-199 or α-MEM without FF (Fig. 1A to E). Without FF, most of these parameters did not differ between TCM-199 and α-MEM, but with FF, they were significantly higher in TCM-199 than in α-MEM, suggesting a synergistic effect between FF and TCM-199. Furthermore, when MAM was conducted in TCM-199 containing FF (199 + FF), percentages of blastocysts and cell counts per blastocyst were significantly higher than those observed in control oocytes. The results suggest that (a) FF in MAM media has profound effects on subsequent maturation and embryo development of pig oocytes; (b) FF and TCM-199 have a synergistic effect on oocyte cytoplasmic maturation; and (c) 199 + FF was the best MAM medium that improved oocyte developmental potential in our experiment.

### MAM with db-cAMP did not improve developmental competence of porcine oocytes

Oocytes were matured for 24 h following MAM for 24 h in 199 + FF containing 25 µM roscovitine or 1 mM db-cAMP. Control oocytes were matured for 48 h without MAM. Although rates of MII oocytes, 2-cell and 4-cell embryos did not differ among treatments, the percentage of blastocysts and the cell number per blastocyst were significantly higher after MAM with roscovitine than with db-cAMP (Fig. 1F). No parameters differed between MAM with db-cAMP and control oocytes.

### Effects of MAM media on post-MAM meiotic progression and cortical granule (CG) redistribution

**Figure 2:**
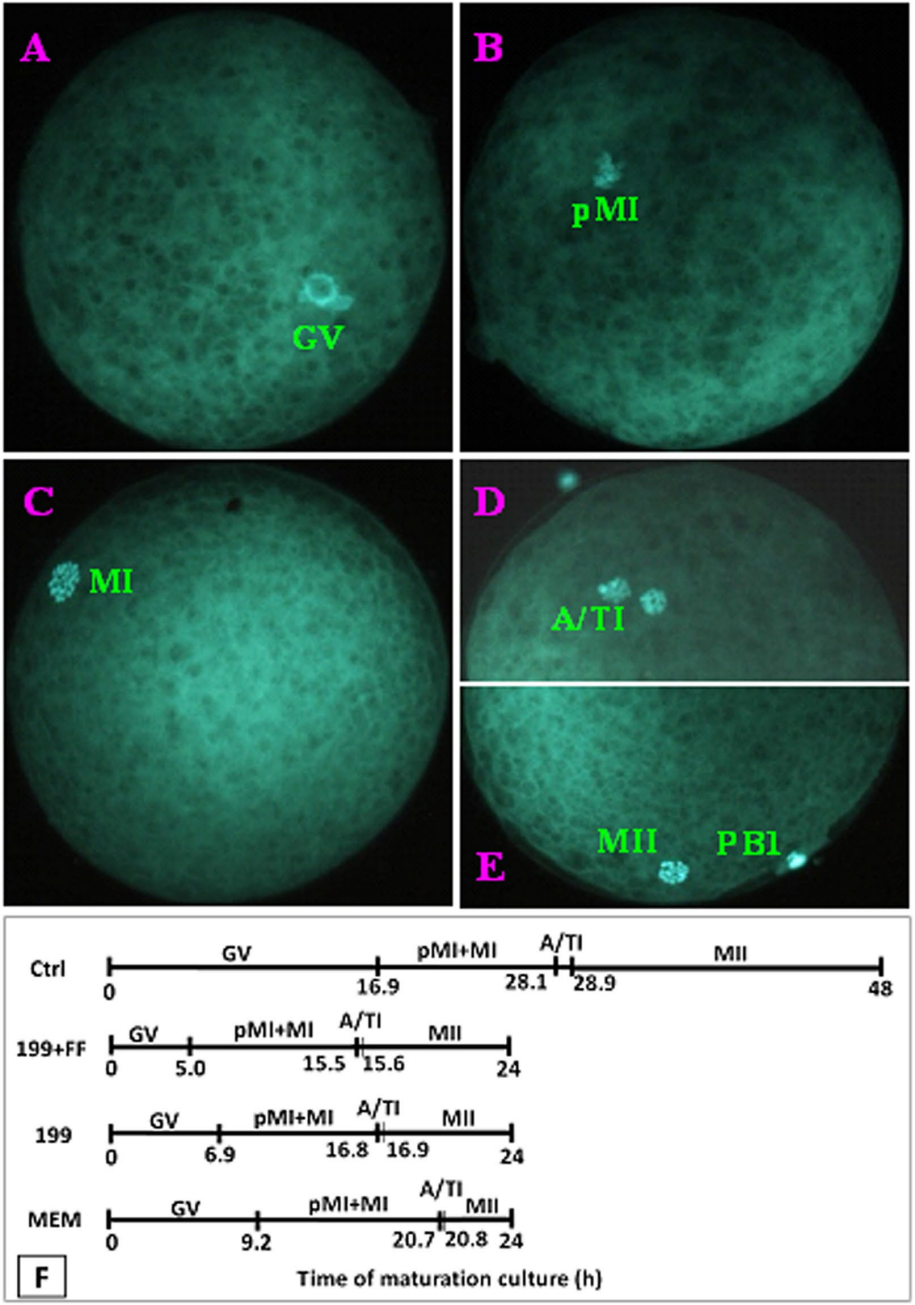
Meiotic progression of pig oocytes during maturation after MAM culture in different MAM media containing 25 µM roscovitine. (**A** to **E**) Fluorescence micrographs of oocytes following Hoechst 33342 staining. Original magnification ×400. (**A**,**B**,**C**,**D** and **E**) show oocytes at the GV, pMI, MI, A/TI and MII stages, respectively. (**F**) The mean time (h) that an oocyte spent at each stage of nuclear progression during maturation without MAM (Ctrl) or after MAM culture in 199 + FF, 199 or MEM. The calculation was done according to Sirard *et al*.^15^ and was based on data collected at 8-h intervals of maturation culture. Each treatment (each MAM medium at each time points) was repeated 4–5 times with each replicate containing about 30 oocytes.

At 8-h and 6-h intervals of the maturation culture following MAM, oocytes were processed to observe nuclear progression and CG redistribution, respectively. To compare the meiotic progression after MAM in different media, oocytes were classified as GV (Fig. 2A), pro-metaphase I (pMI; Fig. 2B), metaphase I (MI; Fig. 2C), anaphase/telophase I (A/TI; Fig. 2D), and MII (Fig. 2E) stages. The average time each stage of nuclear progression lasted was computed using a method reported by Sirard *et al*.^15^. The computation (Fig. 2F) showed that compared to those in non-MAM control oocytes, all the meiotic stages were significantly shortened after MAM in any of the three media, and the shortest ones were observed following MAM in 199 + FF.

**Figure 3:**
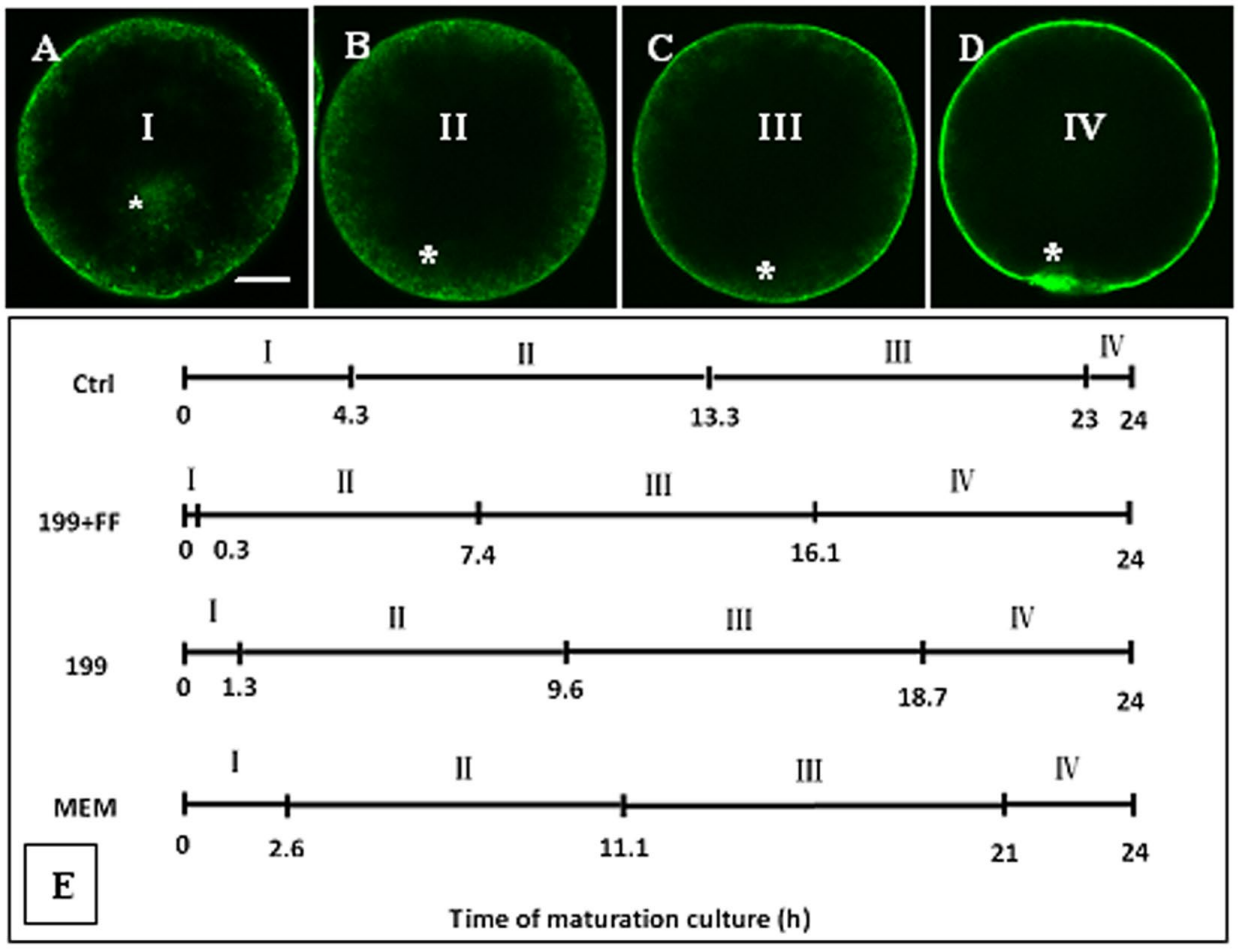
Redistribution of CGs in porcine oocytes at different times of maturation following MAM in different media containing 25 µM roscovitine. Micrographs (**A**,**B**,**C** and **D**) are laser confocal images showing oocytes with I, II, III and VI types of CGs distribution, respectively. The bar is 13 µm and applies to all images. *Indicates the position of the chromatin in the oocyte. (**E**) is a graph showing the mean time (h) that an oocyte spent at each stage of CG redistribution during maturation following MAM in 199 + FF, 199 or MEM medium or in control (Ctrl) oocytes without MAM. Control oocytes were observed during the first 24 h of the maturation culture. The calculation was done according to Sirard *et al*.^15^ and was based on data collected at 6-h intervals of maturation culture. Each treatment (each MAM medium at each time points) was repeated 3–4 times, and each replicate contained 15–20 oocytes.

The distribution of CGs was classified into 4 stages. At stage I, CGs were scattered in the cytoplasm (Fig. 3A); at stage II, CGs were mostly distributed in the cortex with some in the inner cytoplasm (Fig. 3B); at stage III, CGs were mostly in the cortex with some beneath the oolemma (Fig. 3C); and at stage IV, CGs were all located beneath the oolemma (Fig. 3D). The average time each stage of CG redistribution lasted was computed using a method reported by Sirard *et al*.^15^. The computation (Fig. 3E) showed that compared to that in non-MAM control oocytes, the CGs redistribution tempo was significantly quickened after MAM in any of the three media, and the quickest one was observed following MAM in 199 + FF. Taken together, the results suggested that the MAM culture facilitated meiotic progression and CG redistribution of porcine oocytes, and that a quicker post-MAM nuclear progression or CG redistribution, which might suggest a higher cellular activity during MAM, was associated with a higher developmental potential of oocytes, as observed following MAM in 199 + FF.

### Effects of MAM media on cumulus expansion and cumulus cell apoptosis

**Figure 4:**
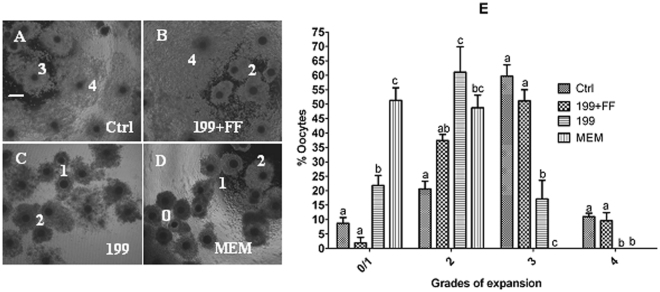
Effects of different MAM media containing 25 µM roscovitine on cumulus expansion. Micrographs (**A**,**B**,**C** and **D)** show cumulus expansion in control oocytes matured for 48 h without MAM (Ctrl) and in oocytes matured for 24 h after MAM in 199 + FF, 199 or MEM, respectively. The bar is 180 µm and applies to all images. The numbers in the pictures indicate grades of cumulus expansion in representative oocytes. (**E**) is a graph showing percentages of oocytes with different grades of cumulus expansion after maturation following MAM in different media. Each treatment was repeated 3–4 times with each replicate including about 30 oocytes. a–c Values without a common letter above their bars differ significantly (P < 0.05) within the same expansion grade.

**Figure 5:**
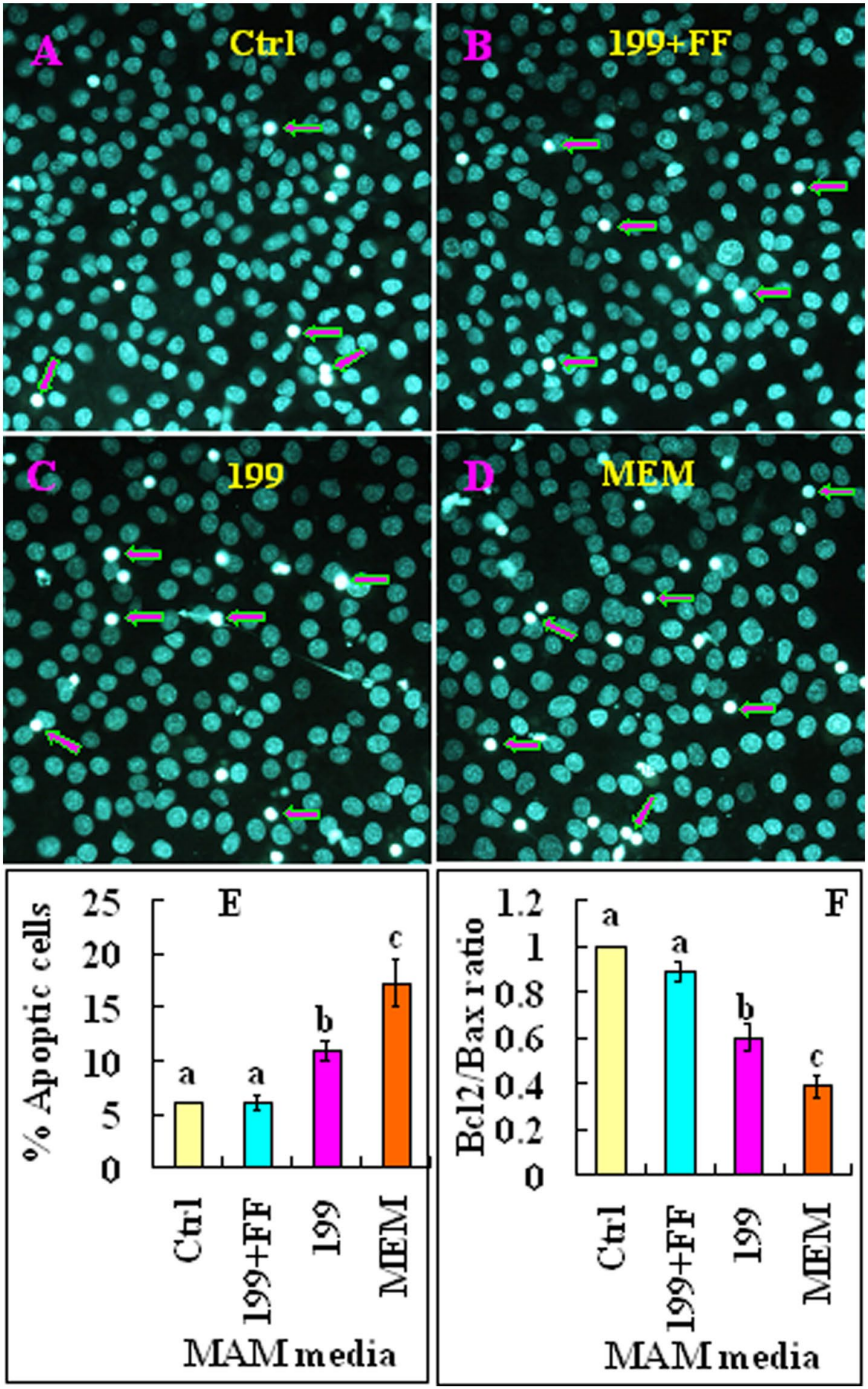
Effects of different MAM media containing 25 µM roscovitine on cumulus cell apoptosis. Micrographs (**A**,**B**,**C** and **D**) show cumulus cells smears from control oocytes matured for 48 h without MAM (Ctrl) or from oocytes matured for 24 h after MAM in 199 + FF, 199 or MEM, respectively. The cumulus cells were stained with Hoechst 33342 before being smeared on a slide and observed under a fluorescence microscope. The heterochromatin was heavily stained with Hoechst, which gave bright fluorescence. Whereas the apoptotic cells showed pyknotic nuclei full of heterochromatin (arrows), healthy cells showed normal nuclei with sparse heterochromatin spots. Original magnification ×400. (**E** and **F**) are graphs showing and Bcl2/Bax mRNA ratio, respectively, in cumulus cells after oocyte maturation following MAM in different media. For calculation of percentages of apoptotic cells, each treatment was repeated 3–4 times with each replicate including 6–8 smears. For PCR analysis of Bcl2 and Bax mRNAs, each treatment was repeated 3–4 times with each replicate containing cumulus cells from 150 oocytes. a–c Values with a different letter above bars differ significantly (P < 0.05).

At the end of the maturation culture following MAM, oocytes were observed for cumulus expansion and cumulus cell apoptosis. Cumulus expansion was scored into 0 to 4 grades. 0 indicates no response; 1 indicates a minimum response, cells in the peripheral two layers began to expand; 2 indicates expansion extended inwards to several layers of cumulus cells; 3 indicates expansion of all layers of the cumulus except corona radiata cells; and 4 indicates expansion of the whole cumulus including corona radiata cells (Fig. 4A–D). Many oocytes from the control and 199 + FF groups showed grade 3 or 4 cumulus expansion (Fig. 4E). However, only a few oocytes from the 199 alone group developed to grade 3, and all the oocytes in the MEM group were blocked at grade 1 or 2 stage of cumulus expansion. In contrast, while the control and 199 + FF oocytes showed a similarly low apoptotic percentage of cumulus cells, percentages of apoptotic cumulus cells increased significantly in the 199 alone and MEM oocytes (Fig. 5). Similarly, while the control and 199 + FF oocytes showed a similarly high Bcl2/Bax mRNA ratio in cumulus cells, the Bcl2/Bax ratio decreased significantly in the 199 alone and MEM oocytes. The results suggested that nutrients (FF) in the MAM medium improved oocyte quality by inhibiting cumulus cell apoptosis while facilitating cumulus expansion.

### Effects of MAM media on levels of intra-oocyte calcium store, ROS and GSH contents

**Figure 6:**
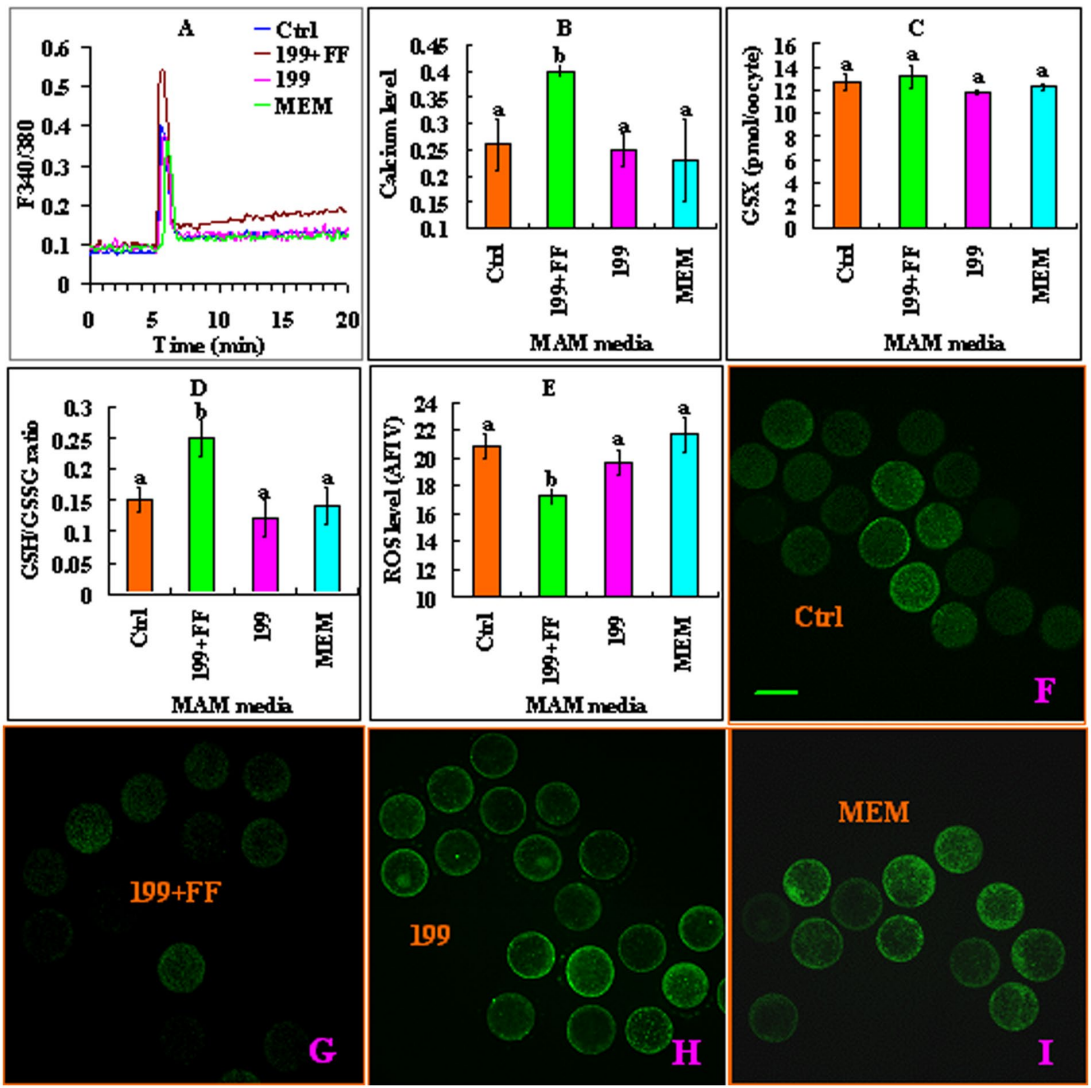
Effects of different MAM media containing 25 µM roscovitine on levels of intra-oocyte calcium, GSH and ROS. Oocytes were matured without MAM (Ctrl) or after MAM in 199 + FF, 199 or MEM. (**A**,**B**,**C**,**D** and **E**) show cytoplasmic calcium profiles (F340/380 ratio), calcium reserves, total glutathione (GSX), GSH/GSSG ratio and ROS levels, respectively. Levels of calcium reserves were calculated by subtracting the basal level from the peak level of calcium. Concentrations of ROS were expressed as average fluorescence intensity value (AFIV) calculated from fluorescence intensity values (FIV) of multiple oocytes. The FIV for each oocyte was calculated by subtracting the basal intensity value from the peak value. a,b: Values with a different letter above bars differ significantly (P < 0.05). (**F**,**G**,**H** and **I**) are confocal images showing ROS levels in different oocytes. Bar is 100 µm.

At the end of maturation culture, the oocytes were processed for the assessment of levels of calcium store and ROS and contents of GSH. Whereas the level of calcium store and the ratio of GSH/GSSG were higher, the level of ROS was lower significantly in oocytes matured after MAM in 199 + FF than in control oocytes matured without MAM and in oocytes matured after MAM in 199 or α-MEM without FF (Fig. 6). The results suggested that FF improved oocyte maturation by increasing calcium store and antioxidant potential during the MAM culture.

### Effects of MAM media on oocyte expression of competence- or apoptosis-related genes

**Figure 7:**
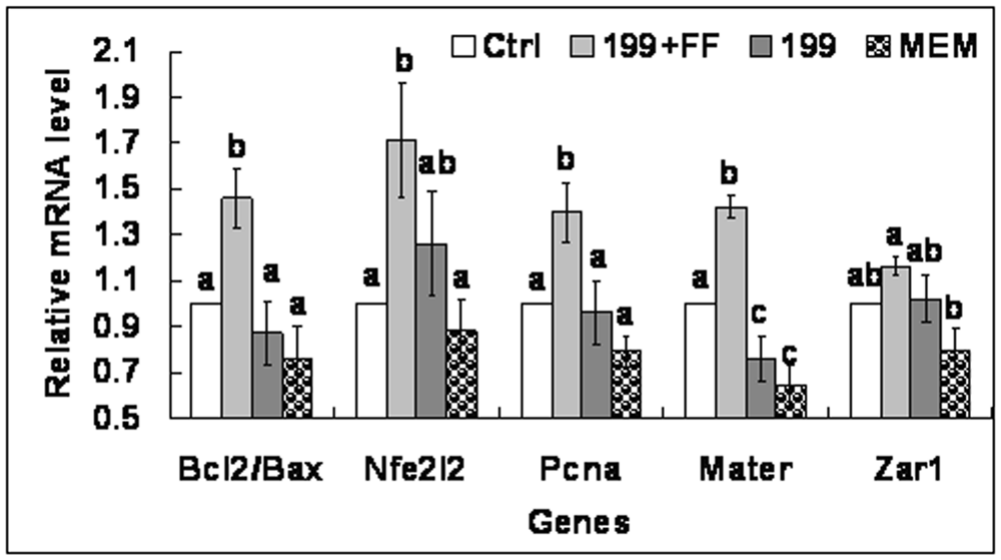
Relative mRNA levels of developmental potential- or apoptosis-related genes in oocytes after maturation following MAM in different media containing 25 µM roscovitine. Values with a different letter above bars differ significantly (p < 0.05). Bcl2, B cell leukemia/lymphoma 2; Bax, BCL2-associated X protein; Nfe2l2, Nuclear factor, erythroid 2 like 2; Pcna, proliferating cell nuclear antigen; Mater, Maternal antigen that embryos require; Zar1, Zygote arrest 1.

At the end of the maturation culture, cumulus-free oocytes were subjected to real-time PCR analysis. The highest level of Bcl2/Bax mRNA ratio, and expression of Nfe2l2, Mater, Zar1 and Pcna mRNAs was observed in oocytes matured after MAM in 199 + FF, and the lowest was in oocytes matured after MAM in MEM alone, with that in control oocytes and oocytes matured after MAM in 199 without FF in between (Fig. 7). The results suggested that FF improved oocyte maturation by promoting the expression of anti-apoptotic genes and genes as predictors of high oocyte developmental competence during the MAM culture.

### Effects of MAM media on oocyte chromatin configuration and gene transcription

**Figure 8:**
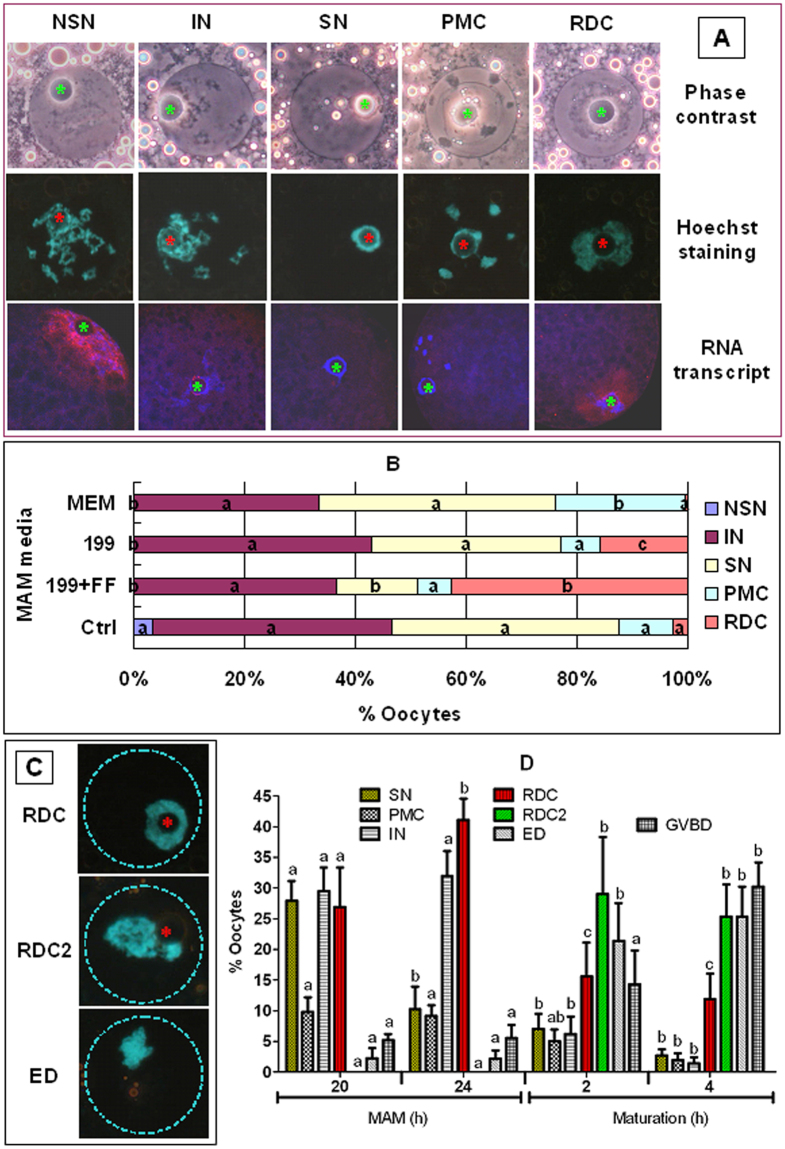
GV chromatin configuration and global RNA transcription in porcine oocytes. Panels A and C show photographs of pig oocytes. Original magnification ×400. ^*^Indicates the location of the nucleolus. In panel A, photographs in the top and middle rows for each chromatin configuration are the same oocyte observed with phase contrast and fluorescence, respectively, after Hoechst 33342 staining. Photographs in the bottom row are laser confocal (merged) images showing global RNA transcription of porcine oocytes with different GV chromatin configurations. DNA and RNA were pseudo colored blue and red, respectively. Original magnification ×630. The chromatin configurations observed include NSN, intermediate (IN), SN, pre-maturely condensed (PMC), re-decondensed (RDC), advanced RDC (RDC2), early diakinesis (ED) and GVBD. Panel B shows percentages of oocytes with different chromatin configurations in control oocytes before MAM culture and in oocytes immediately after a 24-h MAM culture in different media containing 25 µM roscovitine. Each treatment was repeated 5–6 times and each replicate contained 20–30 oocytes. (**A**–**C**) Values with different letters in their bars differ significantly (P < 0.05) in the same configuration. Panel D shows percentages of oocytes with different chromatin configurations in oocytes recovered at 20 or 24 h of MAM culture in 199 + FF containing 25 µM roscovitine or at 2 or 4 h of maturation culture after MAM. Each treatment was repeated 5–6 times and each replicate contained 20–30 oocytes. a-c Values with different letters above bars differ significantly (P < 0.05) within configurations.

Oocytes were examined for chromatin configuration and RNA transcription immediately after MAM culture in different media. Control oocytes were observed immediately after recovery from follicles without MAM. The GV chromatin of porcine oocytes was classified into five configurations, based on the degree and distribution of chromatin condensation (Fig. 8A). The non-surrounded nucleolus (NSN) configuration was characterized by a distinct nucleolus and a diffuse, filamentous pattern of chromatin in the whole GV area. In the surrounded nucleolus (SN) configuration, the nucleolus was surrounded by a complete heterochromatin ring and heterochromatin was not obvious in the nucleoplasm. In the prematurely condensed configuration (PMC), the heterochromatin ring around the nucleolus was often incomplete or forming a horseshoe, and clumps and strands of heterochromatin were observed in the GV. The distribution of heterochromatin in the intermediate (IN) configuration was similar to that in the PMC pattern except that the heterochromatin was only half-condensed. In the re-decondensed (RDC) configuration, the heterochromatin ring around the nucleolus was decondensed into a flocculent pattern.

In the control group before MAM culture, most of the oocytes were at the IN or SN stage (Fig. 8B). Although the proportions of IN and SN oocytes did not change significantly following MAM culture in 199 or MEM media, many SN oocytes took on a RDC configuration after MAM in the 199 + FF medium. Because the occurrence of the RDC configuration prior to GVBD has not been reported before, the origin and fate of this configuration were minutely followed in oocytes recovered at different times before or after the end of MAM in 199 + FF. Three RDC-related configurations were observed including RDC, advanced RDC (RDC2) and early diakinesis (ED) stages (Fig. 8C). The ED stage corresponds to the reported GV4 stage with chromatin condensing into chromosomes and nucleoli disappeared. From 20 h to 24 h of MAM culture, while the percentage of SN oocytes decreased, that of RDC oocytes increased significantly (Fig. 8D). From 24 h of MAM culture to 2 h of maturation culture after MAM, however, while the percentage of RDC oocytes decreased, that of RDC2 and ED oocytes increased significantly. From 2 h to 4 h of maturation culture, the percentage of oocytes undergoing GVBD increased significantly, but little change was observed in other configurations. The results suggest that during maturation, the SN pig oocytes go sequentially through the RDC, RDC2 and ED stages before undergoing GVBD.

Following MAM in 199 + FF containing 1 mM db-cAMP, however, much less oocytes were maintained at the SN stage, no RDC configuration was observed, and the number of RDC2 oocytes increased significantly as the number of IN oocytes decreased from 18 h to 20 h of MAM, compared to those after MAM with roscovitine (Supplementary Fig. S2). The results suggested that MAM with db-cAMP did not induce the RDC configuration at all and that some of the IN oocytes took on the RDC2 configuration directly without going through SN or RDC configuration during MAM with db-cAMP.

Whereas RNA transcription was observed in none of the SN (0/66), PMC oocytes (0/17) or ED (0/24) oocytes, 100% (4/4) of the NSN, 51.1% (23/45) of the RDC, 25% (7/28) of the RDC2 and 15.5% (16/103) of the IN oocytes showed RNA transcribing activities. Taken together, the results suggest that under optimal nutrient conditions during the MAM culture, heterochromatin in some of the SN oocytes was decondensed, and the decondensed chromatin allowed transcription of beneficial genes and rendered the oocytes more developmentally competent. MAM with db-cAMP could not improve oocyte developmental potential because it did not induce the RDC configuration that could promote a substantial gene transcription.

## Discussion

The present results demonstrate that MAM in 199 + FF containing roscovitine not only improved oocyte developmental competence but also facilitated post-MAM meiotic progression, CG redistribution, cumulus expansion, intra-oocyte calcium reserve and the GSH/GSSG ratio while attenuating apoptosis of cumulus cells and oxidative stress of oocytes. Our further analysis showed that MAM in 199 + FF containing roscovitine promoted decondensation of heterochromatin and transcription of beneficial genes in some of the SN oocytes. When MAM was conducted in 199 + FF containing db-cAMP, however, oocyte developmental potential did not improve at all. According to Bagg *et al*.^14^, although treatment of pre-pubertal pig oocytes with db-cAMP increased subsequent blastocyst formation, blastocyst rates of adult oocytes remained unchanged following the same db-cAMP treatment. Because oocytes used in this study were from slaughtered adult sows, the current results have confirmed that 199 + FF containing roscovitine is the suitable MAM medium for IVM of adult pig oocytes.

Oocyte cytoplasmic maturation is often evaluated using multiple markers including CG redistribution, calcium stores, redox state and expression of competence-related genes of oocytes, and cumulus expansion, apoptosis of cumulus cells, as well as preimplantation development^16^. For example, quicker tempos of nuclear maturation and CG redistribution^17–19^ were associated with better cytoplasmic maturation. Bovine oocytes with the highest embryo production efficiency showed greater calcium stores than did the oocytes with a mild embryo production efficiency following IVM^20^. Oxidative stress could influence IVM of oocytes^21^, and cytoplasmic GSH has been defined as an important index for cytoplasmic maturation of porcine oocytes^22, 23^. Furthermore, while an optimal cumulus expansion is essential for normal oocyte maturation and embryo development, a high rate of apoptosis in cumulus cells is associated with impaired oocyte cytoplasmic maturation^24^. Human IVF embryo quality showed a negative correlation with the level of cumulus cell apoptosis^25^.

It is well known that mRNA abundance during oocyte maturation can influence subsequent embryo development^26^. Among the oocyte apoptosis- and developmental potential-related genes analyzed in this study, the ratio of BCL2/BAX determines cell survival or death following an apoptotic stimulus as BCL2 is able to repress apoptosis while overexpressed BAX accelerates apoptotic cell death^27^; NFE2L2 is a transcription factor that enhances cellular protection against oxidative stress^28^; PCNA is a specific marker of the cell cycle S-phase^29^ and an essential component of the DNA replication and repair machinery^30^; and MATER^31^ and ZAR1^32^ are two known oocyte markers required for early embryonic development. Oocytes with a high developmental potential show high levels of Pcna and Nfe2l2 expression following IVM^33, 34^. Developmental arrest of NSN mouse oocyte was associated with reduced expression of MATER and ribosomal proteins^31^. Retrieving oocytes 4 h after slaughter significantly increased the developmental competence of bovine oocytes with higher levels of oocyte Mater and Oct4 transcripts^35^. However, although Zar1 is the first identified oocyte-specific maternal-effect gene that functions at the oocyte-to-embryo transition in the mouse^32^, we could not find any paper supporting a clear positive correlation between levels of Zar1 expression in mature oocytes and subsequent embryo developmental competence in large animals. In contrast, we found a paper reporting no relationship between the relative abundance of Zar1 transcript in zygotes produced under different conditions and the corresponding subsequent embryonic development^36^.

In this study, a new system was adopted for classification of GV chromatin configurations in pig oocytes. Among the new configurations, while the NSN and SN patterns corresponded respectively to the GV0 and GV1 configurations reported previously^37, 38^, the reported GV2 and GV3 configurations were mixed together and classified as the PMC and IN patterns. In addition, the present results showed that MAM in 199 + FF promoted a RDC configuration in many SN oocytes, and the RDC chromatin re-condensed into RDC2 and ED configurations before GVBD. While the RDC configuration supported a significant RNA transcription, the RDC2 and ED oocytes significantly decreased or completely stopped RNA transcription. This is the first report demonstrating that the heterochromatin ring around the nucleolus in a SN oocyte re-decondensed into a flocculent pattern and began RNA transcription *in vitro* under optimal nutrient conditions. It is known that with the growth of GV mouse oocytes, their chromatin configuration changes from NSN to SN pattern. It seems that fully-grown oocytes must end an NSN configuration before gaining full meiotic competence, and they must take on a SN configuration and stop gene transcription before being capable of blastocyst formation^39^. A direct link between the SN configuration and transcription silencing has also been reported in growing oocytes of human beings^40^. However, the GV chromatin is synchronized in a less condensed state prior to GVBD in maturing oocytes of some species that have been minutely studied^39^. For example, the GV chromatin of bovine oocytes was synchronized in a less condensed F pattern with floccular chromatin near the nucleoli and near the nuclear envelope^41^, which is consistent with the observation that mRNA synthesis occurred just prior to GVBD in bovine oocytes^42, 43^. Furthermore, incorporation of [^3^H] uridine by the preovulatory GV oocytes has also been reported in mice^44, 45^ and humans^46^.

This study showed that FF in the MAM medium played a crucial role in promoting gene transcription and improving cytoplasmic maturation of pig oocytes. The beneficial effects of adding FF to maturation media on the developmental competence have been reported in porcine oocytes^47^. Follicular fluid is composed of both transudates from the serum and locally produced molecules, including enzymes, cytokine/growth factors, hormones, energy metabolites and other undefined factors^48, 49^. The concentrations of some of the FF constituents have been demonstrated to correlate with oocyte development, fertilization and early embryo development^49^. For example, FF contains antioxidants such as melatonin and antioxidant enzymes. Melatonin exerted an antioxidant effect protecting oocytes from oxidative stress, and its intra-follicular concentration was inversely correlated with that of the oxidation marker 8OH-deoxyguanosine^50^. Porcine FF contained SOD isoenzymes that efficiently protected oocytes against oxidative stress during IVM through its radical scavenging activity^51^. Furthermore, melatonin accelerates the formation of MPF and GVBD by regulating the maturation-inducing hormone in carp oocytes^52^. Oocyte-secreted factors including BMP-15 and GDF-9 have been detected in porcine FF and have been found to suppress cumulus cell apoptosis and promote the chromatin configuration transition^53, 54^. Maturation in porcine FF significantly increased both cumulus expansion rate and intra-oocyte GSH content of pig oocytes^55^. In addition, the gonadotropin and steroid hormones contained in FF have been reported to promote oocyte maturation. For example, a predominantly estrogenic environment in FF is associated with good follicular growth and has anti-atresia effects, and estrogen has been found to have positive effects on chromatin configuration transition of pig oocytes^53^. Injection of eCG expedited the RNA synthesis of rabbit oocytes to an early and full completion^56^.

In summary, the present results showed that the composition of the MAM media had profound effects on the cytoplasmic maturation of porcine oocytes following IVM, and demonstrated for the first time that MAM in the presence of FF and roscovitine improved oocyte cytoplasmic maturation by promoting a pre-GVBD chromatin re-decondensation and transcription of beneficial genes. Roscovitine, but not db-cAMP, is suitable for MAM of adult pig oocytes. The data are important for improving the IVM systems in both humans and animals.

## Methods

The experimental procedures used for animal care and handling were approved by the Animal Care and Use Committee of the Shandong Agricultural University P. R. China (Permit number: SDAUA-2001-001). The methods were carried out in accordance with the approved guidelines. Unless otherwise specified, all chemicals were purchased from Sigma-Aldrich Corp. (St. Louis, MO, USA).

### Culture media

The medium used for oocyte collection and washing was Dulbecco’s phosphate-buffered saline (D-PBS, HyClone, Logan, UT, USA) supplemented with 0.88 mM CaCl_2_.2H_2_O, 0.49 mM MgCl_2_.6H_2_O, 0.1% polyvinyl alcohol, 0.03 mM phenol red, 50 IU/ml penicillin and 50 µg/ml streptomycin. Four media were used for oocyte MAM. The MEM medium was a simplified α-MEM composed of 1.8 mM CaCl_2_, 0.81 mM MgSO_4_, 5.3 mM KCl, 26.2 mM NaHCO_3_, 117.2 mM NaCl, 1.0 mM NaH_2_PO_4_, 1 mM sodium pyruvate, 2 mM glutamine, 4 mg/ml bovine serum albumin (BSA), 0.03 mM phenol red, 50 IU/ml penicillin, 50 µg/ml streptomycin, 5.56 mM D-glucose, 0.05 IU/ml follicle-stimulating hormone (FSH), and 10 ng/ml epidermal growth factor (EGF). The 199 medium consisted of TCM-199 (Gibco, Grand Island, New York, USA) supplemented with 0.05 IU/ml FSH, 10 ng/ml EGF, 0.57 mM cysteine, 0.1% polyvinyl alcohol, 3.05 mM D -glucose, 0.91 mM sodium pyruvate, 50 IU/ml penicillin and 50 µg/ml streptomycin. The 199 + FF and MEM + FF media were composed of the 199 medium and the MEM medium, respectively, supplemented with 10% porcine follicular fluid (FF). The MAM media were supplemented with different concentrations of roscovitine, db-cAMP or 3-isobutyl-1-methylxanthine (IBMX). The maturation medium used for oocyte maturation culture was the 199 + FF medium supplemented with 0.05 IU/ml luteinizing hormone (LH). The embryo culture medium used was the porcine zygote medium-3 (PZM-3) composed of 108 mM NaCl, 10 mM KCl, 0.35 mM KH_2_PO_4_, 0.4 mM MgSO_4_, 25.07 mM NaHCO_3_, 0.2 mM Na-pyruvate, 2 mM Ca-(lactate)_2_·5H_2_O, 1 mM L-Glutamine, 5 mM Hypotaurine, 20 ml/L essential amino acids, 10 ml/L non-essential amino acids, 0.03 g/L penicillin, 0.02 g/L streptomycin, 4 g/L BSA, pH 7.2–7.4.

Porcine FF was aspirated from 3–6 mm follicles of follicular stage ovaries. After centrifugation at 1,600 × g for 30 min, the supernatants were collected and filtered sequentially through 0.22 µm syringe filters. The prepared FF was then stored at −20 °C until use. To prepare stock solutions, roscovitine (20 mM) and IBMX (200 mM) were dissolved in dimethyl sulfoxide (DMSO), db-cAMP (200 mM) was dissolved in water. The stock solutions were stored in aliquots at −20 °C until use.

### Oocyte preparation and culture

Porcine ovaries were collected from a local abattoir and transported within 3 h after slaughtering to the laboratory at 30–35 °C in 0.9% saline supplemented with 100 IU/ml penicillin and 0.05 mg/ml streptomycin. Oocytes were aspirated from follicles 3–6 mm in diameter using a syringe containing D-PBS. Only oocytes with uniform cytoplasm and compact cumulus were chosen for further use. After washing twice in D-PBS and once in culture medium, 15 to 20 oocytes were cultured in microdrops of 150 µl covered with mineral oil, at 38.5 °C under 5% CO_2_ in humidified air. While oocytes in the control group were cultured in the oocyte maturation medium for 48 h, oocytes in the MAM groups were first cultured for 24 h in corresponding MAM medium (199 + FF, 199 or MEM), and then cultured in the maturation medium for further 24 h. All media were equilibrated at 38.5 °C in an atmosphere of 5% CO_2_ in air for a minimum of 3 h before incubation of oocytes.

### Observation on germinal vesicle (GV) breakdown (GVBD), meiotic progression and GV chromatin configuration

Oocytes were denuded of cumulus cells by pipetting in D-PBS containing 0.1% hyaluronidase. To assess GVBD and meiotic progression, the denuded oocytes were fixed for 2 h in 4% paraformaldehyde before being stained for 10 min at room temperature in D-PBS containing 10 µg/ml of Hoechst 33342. To observe GV chromatin configurations, the denuded oocytes were labeled without fixation for 10 min at 38.5 °C in D-PBS containing 10 µg/ml of Hoechst 33342. The stained oocytes were then placed on glass slides and compressed with coverslips. The mounted oocytes were observed under a Leica DMLB microscope equipped with a CCD camera. Oocytes were first examined with phase contrast to visualize morphology of nucleoli and nuclear envelope, and then observed with fluorescence optics. Hoechst fluorescence was obtained by excitation at 220–360 nm using a mercury lamp (50 W) attenuated with neutral filters.

### Oocyte activation and embryo culture

After maturation culture, oocytes were denuded of cumulus cells as described above. For activation, the denuded oocytes were first treated with 5 µM ionomycin contained in D-PBS for 5 min. Then, the oocytes were washed three times with the PZM-3 medium and incubated for 5 h in PZM-3 medium containing 2 mM 6-DMAP. At the end of the incubation, 6-DMAP was removed by washing the oocytes in D-PBS and the activated oocytes were cultured for embryo development in PZM-3 at 38.5 °C in 5% CO_2_ in air. Cleavage and blastocyst rate were observed at 48 and 168 h of embryo culture, respectively. Cell counts per blastocyst were assessed under a fluorescence microscope after staining for 10 min with 10 µg/ml of Hoechst 33342 in D-PBS.

### Observation on CG redistribution

Zonae pellucidae were removed by treating oocytes with acidic Tyrode solution. After being washed three times in PBS medium, oocytes were fixed with 4% paraformaldehyde in PBS for 30 min at room temperature. The oocytes were then washed three times for 5 min each in PBS medium containing 0.3% BSA and 100 mM glycine. After a 5-min treatment in PBS medium containing 0.1% Triton X-100, oocytes were washed twice for 5 min each in PBS. To label CGs, oocytes were incubated in 100 µg/ml peanut agglutinin labelled with fluorescein isothiocyanate (FITC-PNA) in PBS for 30 min in the dark. After washing three times in PBS containing 0.3% BSA, the oocytes were treated for 10 min with 10 µg/ml of Hoechst 33342 in PBS in the dark to evaluate nuclear status. Finally, the stained oocytes were washed in PBS, mounted on nonfluorescent glass slides and observed with a Leica laser scanning confocal microscope (Leica Micro-systems GmbH, Wetzlar, Germany). Blue diode (405 nm) and argon (488 nm) lasers were used to excite Hoechst and FITC, respectively. Fluorescence was detected with the following bandpass emission filters: 420–480 nm (Hoechst) and 505–540 nm (FITC), and the captured signals were recorded as blue and green, respectively. A single equatorial section was taken from each oocyte, and 15–20 oocytes from each slide were randomly scanned and recorded.

### Assessment of cumulus cells Apoptosis

Hoechst staining was used to assess cumulus cell apoptosis because previous studies have shown that Hoechst staining and TUNEL are comparable methods for detecting apoptosis^57^. The cumulus cells freed from 30–40 oocytes were dispersed by repeatedly pipetting in D-PBS using a thin pipette. The dispersed cells were collected into a 0.5-ml tube and separated from D-PBS by centrifugation (200 × g, 5 min, and room temperature). The cell pellets were then resuspended in 50 µl of D-PBS supplemented with 10 µg/ml of Hoechst 33342 and stained in the dark for 5 min. The stained cells were then centrifuged (200 × g, 5 min, and room temperature) again to concentrate cells. After removal of approximately half the supernatant, a 5 µl drop of suspension was smeared on the slide and observed under a Leica DMLB fluorescence microscope. Six to eight fields were randomly examined on each smear, and percentages of apoptotic cells were calculated from at lest 60–80 cells observed in each field. The percentages of apoptotic cells were calculated double blindly by two investigators.

### Measurement for calcium stores

Cumulus-free oocytes were incubated at 38.5 °C for 30 min in PZM-3 medium containing 1 µM Fura-2 AM and 0.02% pluronic F-127 to load the Ca^2+^ probe. Oocytes were then transferred into a PZM-3 drop in Fluoro dish (FD35–100, World Precision Instruments), covered with mineral oil, and observed at 38.5 °C with a Leica DMI6000 inverted microscope. The fluorescence was excited using a Fura 2 fluorescence module, and the F340/380 ratio was calculated using a Leica LAS-AF calcium imaging module. The oocytes were monitored for 5 min to record the baseline F340/380 ratio, and then, ionomycin was added to the PZM-3 drop to give a final concentration of 50 µM. After ionomycin addition, the oocytes were monitored for 20 min to record the peak F340/380 ratio. While the baseline F340/380 ratio represented the cytoplasmic calcium, the difference between the peak and baseline F340/380 ratios represented the calcium stores of an oocyte.

### Assay for intra-oocyte reactive oxygen species (ROS)

Intra-oocyte H_2_O_2_ levels were measured using 2′,7′-dichlorodihydrofluorescein diacetate (DCHFDA). A stock solution of 1 mM DCHFDA was prepared in DMSO and stored in the dark at −20 °C. The stock solution was diluted to 0.01 mM with D-PBS immediately before use, and cumulus-free oocytes were stained for 10 min with the resultant DCHFDA solution. After a thorough washing, oocytes were placed on a slide, and observed under a Leica laser scanning confocal microscope. Fluorescence was obtained by excitation at 488 nm, and photographs were taken using fixed microscopic parameters. The fluorescence intensity from each oocyte was analyzed using a Leica software.

### Measurement of intraoocyte GSH

After cumulus-free oocytes were washed three times in Ca^2+^-, Mg^2+^-free PBS medium, 5 µl of distilled water containing 20–30 oocytes were transferred to a 1.5-ml tube. Then, 5 µl of 1.25 M phosphoric acid were added to the tube. Following frozen at −80 °C and thawed at room temperature three times, the samples were stored at −70° until analysis. A 5,5′-dithio-bis (2-nitrobenzoic acid) (DTNB)-oxidized GSH (GSSG) reductase-recycling assay was performed to determine the total GSH (GSX) concentration. Briefly, 700 µl of 0.33 mg/ml of NADPH in a stock buffer (0.2 M sodium phosphate buffer containing 10 mM EDTA, pH 7.2), 100 µl of 6 mM DTNB in the stock buffer, and 190 µl of distilled water were added to the sample tube. Then, 10 µl of GSH reductase (G-3664; 250 IU/ml) were added with mixing to initiate the reaction. The absorbance was monitored continuously for 3 min with a spectrophotometer at 412 nm, with reading recorded every 0.5 min. To measure GSSG concentrations, 10 µl samples were vigorously mixed with 0.2 µl of 2-vinylpyridine and 0.6 µl of triethanolamine. Sixty minutes later, the samples were assayed as described above in the DTNB-GSSG reductase-recycling assay. GSX standards (0.01, 0.02, 0.1, 0.2, and 1.0 mM) and a sample blank were also assayed. To calculate the intracellular GSX concentration per oocyte (pmol/oocyte), the amount of GSX was divided by the number of oocytes in each sample. The reduced GSH (GSH) values for each oocyte were calculated from the difference between GSX and GSSG.

### Real-time RT-PCR

RNA was extracted from 300 cumulus-free oocytes or the cumulus cells removed from 150 oocytes using a commercial RNA isolation kit (RNAqueous-Micro Kit, cat. no. AM1931). A total volume of 20 µl was used to perform reverse transcription. Thus, 2 µl of each RNA sample was mixed with 1 µl of Oligo(dT)18 (Fermentas) and 10 µl of DEPC-dH_2_O in a 0.2 ml reaction tube, and the mixture was incubated in a PCR instrument at 65 °C for 10 min. At the end of the incubation period, the reaction tube was cooled on ice for 2 min, and then, 4 µl of 5× reverse transcriptase (RT) buffer, 2 µl of deoxynucleotide (dNTP), 0.5 µl of RNase inhibitor, and 0.5 µl of Superscript III Reverse Transcriptase (Roche) were added to the reaction tube. The mixture was incubated at 55 °C for 30 min, at 85 °C for 5 min, and stored at −20 °C until use.

Gene-specific primers for real-time RT-PCR are listed in Supplementary Table S1. A Mx3005 P Real-Time PCR System (Stratagene) was used for mRNA quantification. a 10 µl reaction volume was used for the amplification reactions including 1 µl of cDNA, 5 µl of 2× SYBR Green Master Mix (Agilent), 0.15 µl of 500-fold diluted reference dye, 3.25 µl of RNase-free water, and 0.3 µl each of forward and reverse gene-specific primers (10 μM). The cycle amplification conditions: (1) an initial denaturation step at 95 °C for 3 min; (2) 40 cycles at 95 °C for 20 sec; and (3) annealing temperature for 20 sec. To determine specificity of the reaction, PCR products were analyzed by sequencing, dissociation curve analysis, and gel electrophoresis. The expression of each gene was evaluated based on the β-actin expression. All values were then expressed relative to calibrator samples (control oocytes) using the 2^−(ΔΔT)^ method.

### Detection of global RNA transcription

Oocytes were labeled for 2 h in 100 µl BCM containing 1 mM 5-ethynyl uridine (EU) at 38.5 °C under 5% CO_2_ in humidified air. All the steps of EU detection were performed at room temperature according to the manufacturer’s instructions (Invitrogen; Click-iT RNA imaging kits). After EU labeling, oocytes were (1) freed of cumulus and zona pellucida; (2) fixed using 3.7% formaldehyde in PBS for 40 min; (3) permeabilized with 0.1% Triton X-100 for 30 min; (4) stained for 30 min with 100 mM Tris (pH 8.5)/1 mM CuSO4/10–50 µM fluorescent azide/100 mM ascorbic acid, protected from light; (5) washed with Click-iT® reaction rinse buffer; (6) stained with Hoechst 33342; and (7) mounted on glass slides and observed with a Leica laser scanning confocal microscope (TCS SP2; Leica Microsystems). Blue diode (405 nm) and argon (Ar; 488 nm) lasers were used to excite Hoechst and FITC, respectively. Fluorescence was detected with the following bandpass emission filters: 420–480 nm for Hoechst and 505–540 nm for FITC.

### Data analysis

Each treatment was repeated at least three times. Percentage data were arc sine transformed before being analyzed with ANOVA. A Duncan multiple comparison test was used to find differences. The software of Statistics Package for Social Sciences (SPSS 11.5, SPSS Inc. Chicago, IL) was used. Data were expressed as mean ± SEM, and P < 0.05 was considered significant.

## Electronic supplementary material

Zhang et al. Supplementary information

## References

[1] Hyttel P, Fair T, Callesen H, Greve T (1997). Oocyte growth, capacitation and final maturation in cattle. Theriogenology..

[2] Wu GM (2002). High developmental competence of pig oocytes after meiotic inhibition with a specific M-phase promoting factor kinase inhibitor, butyrolactone I. Biol. Reprod..

[3] Bilodeau-Goeseels S (2012). Bovine oocyte meiotic inhibition before *in vitro* maturation and its value to *in vitro* embryo production: does it improve developmental competence?. Reprod. Domest. Anim..

[4] Funahashi H, Cantley TC, Day BN (1997). Synchronization of meiosis in porcine oocytes by exposure to dibutyryl cyclic adenosine monophosphate improves developmental competence following *in vitro* fertilization. Biol. Reprod..

[5] Hashimoto S, Minami N, Takakura R, Imai H (2002). Bovine immature oocytes acquire developmental competence during meiotic arrest *in vitro*. Biol. Reprod..

[6] Zeng HT (2013). Heparin and cAMP modulators interact during pre-*in vitro* maturation to affect mouse and human oocyte meiosis and developmental competence. Hum. Reprod..

[7] Zeng HT (2014). Prematuration with cyclic adenosine monophosphate modulators alters cumulus cell and oocyte metabolism and enhances developmental competence of *in vitro*-matured mouse oocytes. Biol. Reprod..

[8] Ezoe K (2015). Developmental competence of vitrified-warmed bovine oocytes at the germinal-vesicle stage is improved by cyclic adenosine monophosphate modulators during *in vitro* maturation. PLoS One..

[9] Romero S, Sánchez F, Lolicato F, Van Ranst H, Smitz J (2016). Immature oocytes from unprimed juvenile mice become a valuable source for embryo production when using C-type natriuretic peptide as essential component of culture Medium. Biol. Reprod..

[10] Buell M, Chitwood JL, Ross PJ (2015). cAMP modulation during sheep in vitro oocyte maturation delays progression of meiosis without affecting oocyte parthenogenetic developmental competence. Anim. Reprod. Sci..

[11] Sirard MA, First NL (1988). *In vitro* inhibition of oocyte nuclear maturation in the bovine. Biol. Reprod..

[12] Ma S (2003). Hypoxanthine (HX) inhibition of *in vitro* meiotic resumption in goat oocytes. Mol. Reprod. Dev..

[13] Bilodeau-Goeseels S (2011). Cows are not mice: the role of cyclic AMP, phosphodiesterases, and adenosine monophosphate-activated protein kinase in the maintenance of meiotic arrest in bovine oocytes. Mol. Reprod. Dev..

[14] Bagg MA, Nottle MB, Grupen CG, Armstrong DT (2006). Effect of dibutyryl cAMP on the cAMP content, meiotic progression, and developmental potential of *in vitro* matured pre-pubertal and adult pig oocytes. Mol. Reprod. Dev..

[15] Sirard MA (1989). Timing of nuclear progression and protein synthesis necessary for meiotic maturation of bovine oocytes. Biol. Reprod..

[16] Watson AJ (2007). Oocyte cytoplasmic maturation: a key mediator of oocyte and embryo developmental competence. J. Anim. Sci..

[17] Dominko T, First NL (1997). Timing of meiotic progression in bovine oocytes and its effect on early embryo development. Mol. Reprod. Dev..

[18] Wang Q, Sun QY (2007). Evaluation of oocyte quality: morphological, cellular and molecular predictors. Reprod. Fertil. Dev..

[19] Liu XY (2005). Cortical granules behave differently in mouse oocytes matured under different conditions. Hum. Reprod..

[20] Boni R, Cuomo A, Tosti E (2002). Developmental potential in bovine oocytes is related to cumulus-oocyte complex grade, calcium current activity, and calcium stores. Biol. Reprod..

[21] Combelles CM, Gupta S, Agarwal A (2009). Could oxidative stress influence the *in-vitro* maturation of oocytes?. Reprod. Biomed. Online..

[22] Meister A, Anderson ME, Hwang O (1986). Intracellular cysteine and glutathione delivery systems. J.Am. Coll. Nutr..

[23] Funahashi H, Cantley TC, Stumpf TT, Terlouw SL, Day BN (1994). Use of low-salt culture medium for *in vitro* maturation of porcine oocytes is associated with elevated oocyte glutathione levels and enhanced male pronuclear formation after *in vitro* fertilization. Biol. Reprod..

[24] Arias-Álvarez M (2016). *In vivo* and *in vitro* maturation of rabbit oocytes differently affects the gene expression profile, mitochondrial distribution, apoptosis and early embryo development. Reprod. Fertil. Dev..

[25] Corn CM, Hauser-Kronberger C, Moser M, Tews G, Ebner T (2005). Predictive value of cumulus cell apoptosis with regard to blastocyst development of corresponding gametes. Fertil. Steril..

[26] Lonergan P (2003). Relative messenger RNA abundance in bovine oocytes collected *in vitro* or *in vivo* before and 20 hr after the preovulatory luteinizing hormone surge. Mol. Reprod. Dev..

[27] Oltvai ZN, Milliman CL, Korsmeyer SJ (1993). Bcl-2 heterodimerizes *in vivo* with a conserved homolog, Bax, that accelerates programmed cell death. Cell..

[28] Miao W, Hu L, Scrivens PJ, Batist G (2005). Transcriptional regulation of NF-E2 p45-related factor (NRF2) expression by the aryl hydrocarbon receptor-xenobiotic response element signaling pathway: direct cross-talk between phase I and II drug-metabolizing enzymes. J. Biol. Chem..

[29] Leibovici M, Monod G, Géraudie J, Bravo R, Méchali M (1992). Nuclear distribution of PCNA during embryonic development in Xenopus laevis: a reinvestigation of early cell cycles. J. Cell Sci..

[30] Kelman Z (1997). PCNA: structure, functions and interactions. Oncogene.

[31] Monti M (2013). Developmental arrest and mouse antral not-surrounded nucleolus oocytes. Biol. Reprod..

[32] Wu X (2003). Zygote arrest 1 (Zar1) is a novel maternal-effect gene critical for the oocyte-to-embryo transition. Nat. Genet..

[33] Yoon JD (2015). Effects of coculture with cumulus-derived somatic cells on *in vitro* maturation of porcine oocytes. Theriogenology..

[34] Jiao GZ (2016). Optimized protocols for in vitro maturation of rat oocytes dramatically improve their developmental competence to a level similar to that of ovulated oocytes. Cell Reprogram..

[35] Urrego R (2015). Follicular progesterone concentrations and messenger RNA expression of MATER and OCT-4 in immature bovine oocytes as predictors of developmental competence. Theriogenology..

[36] Shirazi A, Motaghi E (2013). The in vitro fertilization of ovine oocytes in the presence of oviductal cells and its effect on the expression of zygote arrest 1 (Zar1) and subsequent embryonic development. J. Reprod. Infertil..

[37] Motlik J, Fulka J (1976). Breakdown of the germinal vesicle in pig oocytes *in vivo* and *in vitro*. J. Exp. Zool..

[38] Sun XS, Liu Y, Yue KZ, Ma SF, Tan JH (2004). Changes in germinal vesicle (GV) chromatin configurations during growth and maturation of porcine oocytes. Mol. Reprod. Dev..

[39] Tan JH (2009). Chromatin configurations in the germinal vesicle of mammalian oocytes. Mol. Hum. Reprod..

[40] Miyara F (2003). Chromatin configuration and transcriptional control in human and mouse oocytes. Mol. Reprod. Dev..

[41] Liu Y (2006). Germinal vesicle chromatin configurations of bovine oocytes. Microsc. Res. Tech..

[42] Hunter AG, Moor RM (1987). Stage-dependent effects of inhibiting ribonucleic acids and protein synthesis on meiotic maturation of bovine oocytes *in vitro*. J. Dairy. Sci..

[43] Kastrop PM, Hulshof SC, Bevers MM, Destree OH, Kruip TA (1991). The effects of a-amanitin and cycloheximide on nuclear progression, protein synthesis, and phosphorylation during bovine oocyte maturation *in vitro*. Mol. Reprod. Dev..

[44] Wassarman PM, Letourneau GE (1976). RNA synthesis in fully-grown mouse oocytes. Nature..

[45] Kopecny V, Landa V, Pavlok A (1995). Localization of nucleic acids in the nucleoli of oocytes and early embryos of mouse and hamster: An autoradiographic study. Mol. Reprod. Dev..

[46] Tesarik J, Kopecny V, Kurilo LF (1984). Pre-ovulatory RNA synthesis in human oocytes of large antral follicles. Histochem. J..

[47] Ito M (2008). Effect of follicular fluid collected from various diameter follicles on the progression of nuclear maturation and developmental competence of pig oocytes. Anim. Reprod. Sci..

[48] Fortune JE (1994). Ovarian follicular growth and development in mammals. Biol. Reprod..

[49] Revelli A (2009). Follicular fluid content and oocyte quality: from single biochemical markers to metabolomics. Reprod. Biol. Endocrinol..

[50] Tamura H (2008). Oxidative stress impairs oocyte quality and melatonin protects oocytes from free radical damage and improves fertilization rate. J. Pineal. Res..

[51] Tatemoto H, Muto N, Sunagawa I, Shinjo A, Nakada T (2004). Protection of porcine oocytes against cell damage caused by oxidative stress during *in vitro* maturation: role of superoxide dismutase activity in porcine follicular fluid. Biol. Reprod..

[52] Chattoraj A (2005). Melatonin accelerates maturation inducing hormone (MIH): induced oocyte maturation in carps. Gen. Comp. Endocrinol..

[53] Sun MJ (2016). An essential role for the intra-oocyte MAPK activity in the NSN-to-SN transition of germinal vesicle chromatin configuration in porcine oocytes. Sci. Rep..

[54] Han X (2017). MicroRNA-21 plays a pivotal role in the oocyte-secreted factor-induced suppression of cumulus cell apoptosis. Biol. Reprod..

[55] Bijttebier J, Van Soom A, Meyer E, Mateusen B, Maes D (2008). Preovulatory follicular fluid during *in vitro* maturation decreases polyspermic fertilization of cumulus-intact porcine oocytes *in vitro* maturation of porcine oocytes. Theriogenology..

[56] Wang HL (2009). Dynamic changes of germinal vesicle chromatin configuration and transcriptional activity during maturation of rabbit follicles. Fertil. Steril..

[57] Eldadah BA, Ren RF, Faden AI (2000). Ribozyme-mediated inhibition of caspase-3 protects cerebellar granule cells from apoptosis induced by serum-potassium deprivation. J. Neurosci..

