# Supplementary material for: Meiotic arrest with roscovitine and follicular fluid improves cytoplasmic maturation of porcine oocytes by promoting chromatin de-condensation and gene transcription
Source: Sci Rep. 2017 Sep 14;7:11574. doi: 10.1038/s41598-017-11970-y (PMC5599650; doi:10.1038/s41598-017-11970-y)
Supplement: Supplementary file 1 — Zhang et al. Supplementary information [file 41598_2017_11970_MOESM1_ESM.pdf]

Supplementary information

Title: Meiotic arrest with roscovitine and follicular fluid improves cytoplasmic maturation of porcine oocytes by promoting chromatin de-condensation and gene expression

Min Zhang[1,2], Chuan-Xin Zhang[1,2], Liu-Zhu Pan[1], Shuai Gong[1], Wei Cui[1], Hong-Jie Yuan[1], Wei-Ling Zhang[1] and Jing-He Tan[1,3]

1. College of Animal Science and Veterinary Medicine, Shandong Agricultural University, Tai-an City 271018, P. R. China

2. These authors contributed equally to this work.

3. Correspondence: Jing-He Tan, Ph.D., College of Animal Science and Veterinary Medicine, Shandong Agricultural University, Tai-an City, Shandong Province, P R China, Post code: 271018, Phone: 0538-8249616, FAX: 0538-8241419, Email: tanjh@sdau.edu.cn

Supplementary figure and table legends

Supplementary Figure S1. Percentages of GV-intact oocytes after MAM for 24 h in the MEM medium containing different concentrations of roscovitine, db-cAMP or IBMX. Each treatment was repeated 4-5 times with each replicate including about 30 oocytes. a-c: Values with a different letter above bars differ significantly ($P<0.05$).

Supplementary Figure S2. Percentages of oocytes with different chromatin configurations after MAM culture in 199+FF containing 1 mM db-cAMP for 18 h or 20 h or roscovitine for 20 h or 24 h. Each treatment was repeated 5-6 times and each replicate contained 20-30 oocytes. a-c: Values with different letters above bars differ significantly ($P<0.05$) within configurations.

Supplementary Table S1. Oligonucleotide primer sequences used for real-time PCR in this study.

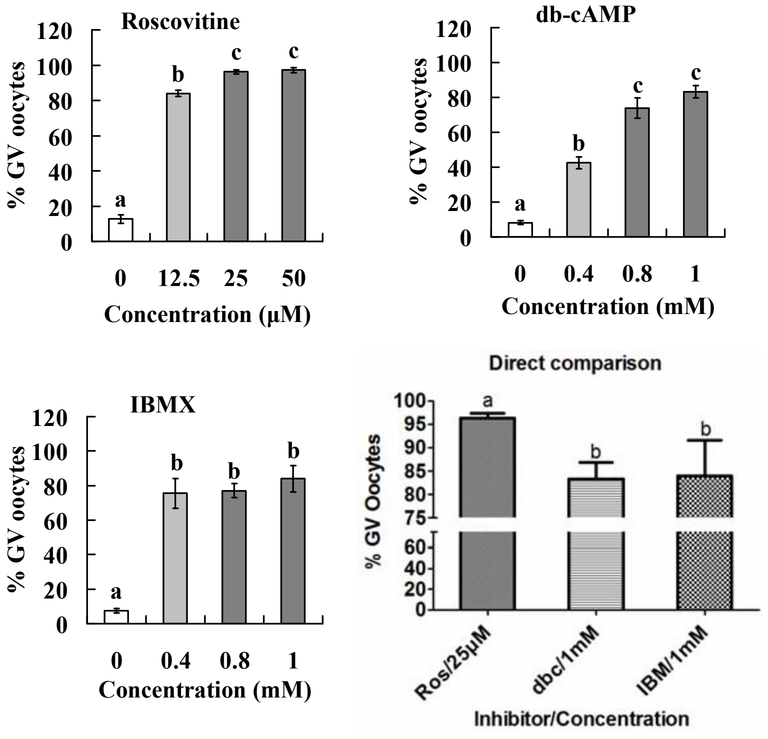

Supplementary Figure S1. Percentages of GV-intact oocytes after MAM for 24 h in the MEM medium containing different concentrations of roscovitine, db-cAMP or IBMX. Each treatment was repeated 4-5 times with each replicate including about 30 oocytes. a-c: Values with a different letter above bars differ significantly ($P<0.05$).

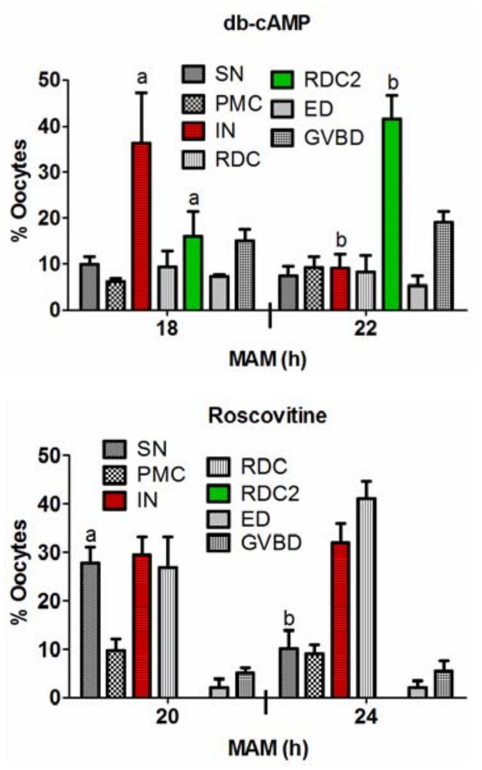

Supplementary Figure S2. Percentages of oocytes with different chromatin configurations after MAM culture in 199+FF containing 1 mM db-cAMP for 18 h or 20 h or roscovitine for 20 h or 24 h. Each treatment was repeated 5-6 times and each replicate contained 20-30 oocytes. a-c: Values with different letters above bars differ significantly (P<0.05) within configurations.

Supplementary Table S1. Oligonucleotide primer sequences used for real-time PCR in this study

cDNA	Oligonucleotide sequences (5’-3’)	Amplified product size (bp)	Annealing temperature (°C)
β-actin	F: CGTGCGGGACATCAAGGA<br>R: AGGAAGGAGGGCTGGAAGA	177	—
Pcna	F: AGAGGAGGAAGCAGTTACCAT<br>R: CTGTAGGAGAGAGTGGAGTGG	104	58
Bax	F: CCAAGAAGTTGAGCGAGTGTC<br>R: CGTCCCAAAGTAGGAGAGGA	335	58
Bcl2	F: AGGGCATTCAGTGACCTGAC<br>R: CGATCCGACTCACCAATACC	193	58
Nfe2l2	F: CCCATTCACAAAAGACAAACATTC<br>R: GCTTTTGCCCTTAGCTCATCTC	72	58
Mater	F: AGCATCTCACCTCCCTCTTG<br>R: AATCAATCCCTTCCACCTCA	116	60
Zar1	F: TGGTGTGTCCAGGGCACTAA<br>R: GTCACAGGAGAGGCGTTTGC	213	62

F, Forward; R, Reverse.
